# Sleep disturbances are associated with cortical and subcortical atrophy in alcohol use disorder

**DOI:** 10.1038/s41398-021-01534-0

**Published:** 2021-08-16

**Authors:** Rui Zhang, Dardo Tomasi, Peter Manza, Ehsan Shokri-Kojori, Sukru B. Demiral, Dana E. Feldman, Danielle S. Kroll, Catherine L. Biesecker, Katherine L. McPherson, Gene-Jack Wang, Corinde E. Wiers, Nora D. Volkow

**Affiliations:** 1 National Institute on Alcohol Abuse and Alcoholism, Laboratory of Neuroimaging, National Institutes of Health, Bethesda, MD 20892-1013 USA; 2 Walter Reed Army Institute of Research, 503 Robert Grant Ave, Silver Spring, MD 20910 USA; 3 National Institute on Drug Abuse, National Institutes of Health, Bethesda, MD 20892-1013 USA

**Keywords:** Neuroscience, Addiction

## Abstract

Sleep disturbances are prominent in patients with alcohol use disorder (AUD) and predict relapse. So far, the mechanisms underlying sleep disruptions in AUD are poorly understood. Because sleep-related regions vastly overlap with regions, where patients with AUD showed pronounced grey matter (GM) reduction; we hypothesized that GM structure could contribute to sleep disturbances associated with chronic alcohol use. We combined sleep EEG recording and high-resolution structural brain imaging to examine the GM-sleep associations in 36 AUD vs. 26 healthy controls (HC). The patterns of GM-sleep associations differed for N3 vs. REM sleep and for AUD vs. HC. For cortical thickness (CT), CT-sleep associations were significant in AUD but not in HC and were lateralized such that lower CT in right hemisphere was associated with shorter N3, whereas in left hemisphere was associated with shorter REM sleep. For the GM density (GMD), we observed a more extensive positive GMD-N3 association in AUD (right orbitofrontal cortex, cerebellum, dorsal cingulate and occipital cortex) than in HC (right orbitofrontal cortex), and the GMD-REM association was positive in AUD (midline, motor and paralimbic regions) whereas negative in HC (the left supramarginal gyrus). GM structure mediated the effect of chronic alcohol use on the duration of N3 and the age by alcohol effect on REM sleep. Our findings provide evidence that sleep disturbances in AUD were associated with GM reductions. Targeting sleep-related regions might improve sleep in AUD and enhance sleep-induced benefits in cognition and emotional regulation for recovery.

## Introduction

Sleep disturbances are highly prevalent in alcohol use disorder (AUD) [1]. Both N3 (slow-wave sleep) and rapid-eye-movement (REM) sleep are altered in AUD and only limited sleep improvement is observed within the first 30 days of abstinence [2]. Sleep disturbances increase the risk of relapse in AUD [3, 4] and failed sleep treatment interferes with abstinence [2], highlighting the need to understand the mechanisms underlying sleep disturbances in AUD.

Slow-wave sleep (SWS) and REM sleep involve distinct brain regions. Slow waves occur locally and then propagate across the cortex, particularly the frontal and temporal lobes [5–7]. In contrast, during REM sleep parietal and occipital visual attentional areas [8–10] and limbic regions [10, 11] are greatly activated. There are also regions shared by SWS and REM sleep: the midline cingulate regions, which is a core component of the default mode network (DMN), not only spreads slow waves from anterior to posterior brain areas but also activated during REM sleep [5, 6, 9, 11].

Intriguingly, these sleep-related regions vastly overlap with regions where patients with AUD showed prominent gray matter (GM) reduction [12]. Lower GM density (GMD) and cortical thickness (CT) in AUD are most prominent in frontal lobe subregions (e.g., superior frontal gyrus, precentral gyrus), midline DMN (e.g., medial orbitofrontal cortex [OFC], anterior cingulate cortex [ACC] and precuneus), inferior parietal cortex (especially supramarginal gyrus), temporal gyrus, limbic regions (e.g., entorhinal and parahippocampal cortex, amygdala, and insula) and occipital cortex [12–16]. GM reductions in many of these regions are associated with less SW activity (SWA) and/or density in healthy adolescents and adults [17–19]. Although direct evidence for a relationship between REM sleep and GM structure is sparse, patients with REM behavioral disorders (RBD) have diminished CT and GMD in frontal (e.g., medial superior frontal, OFC and ACC), postcentral gyrus, medial temporal, and occipital regions compared to healthy controls (HC) [20–22]. So far, the link between sleep (SWS and REM) and GM in AUD has not yet been investigated. Therefore, we examined whether sleep disturbances in AUD, particularly in N3 and REM sleep, were associated with GM differences as compared to healthy social drinkers. We examined both GMD and CT because they provide different information about changes in GM structure and do not always correspond to each other [23, 24]. We hypothesized that GM reductions would mediate the effect of chronic alcohol use on SWS and REM sleep in AUD.

## Materials and methods

### Participants

Data of 36 AUDs and 26 HCs were used in the current analyses. See Supplementary section “Participants: inclusion and exclusion criteria”. AUDs and HCs did not differ in age and gender (Table 1). Clinical and demographic information of participants were assessed on the first day of study (Table 1).

**Table 1:** Clinical, demographic, and sleep information for AUD and HC.

Characteristic	AUD (*N* = 36)	HC (*N* = 26)	*P*-value
	Mean (SD)	Mean (SD)	
Age	41.51 (13.21)	40.53 (13.13)	0.774
Gender (Female %)	27.8	46.2	0.136
Race (%)	African–American 38.9 White 47.4 Asian 0 Multiracial 2.8 Unknown 11.1	African–American 53.8 White 34.6 Asian 3.8 Multiracial 2.8 Unknown 3.8	0.441
ADS	21.40 (8.65)	0.11 (.43)	<**0.001**
AUDIT	29.43 (6.33)	1.46 (1.33)	<**0.001**
Maximum CIWA at admission	4.86 (2.94)	–	–
LDH: Total lifetime drinks	80663.02 (82904.01)	1472.17 (3254.03)	<**0.001**
LDH: Years of heavy drinking	13.80 (8.70)	0.27 (1.37)	<**0.001**
LDH: Age of first drink	15 (6.90)	18.29 (3.20)	0.068
Years of alcohol use	26.97 (13.93)	5.70 (8.30)	<**0.001**
TLFB 90 days (drinks/day)	15.23 (9.05)	0.90 (0.92)	<**0.001**
Type of Benzo use (% of patients)	Diazepam 8.3 Oxazepam 63.9 No use 27.8	–	–
Average dose of Benzo use on 1st WK (mg Oxazepam)^a^	17.02 (27.86)		
Smokers (%)	52.8	0	<**0.001**
FTND	3.95 (1.87)	–	–
BDI	13.78 (9.20)	1.13 (3.02)	<**0.001**
STAI anxiety	50.43 (11.85)	25.35 (5.76)	<**0.001**
Caffeine use (mg/day)	29.08 (94.32)	17.15 (52.00)	0.562
*Sleep parameter*			
TST (h)	4.69 (1.23)	7.50 (1.41)	<**0.001**
N2 (h)/(% of TST)	2.64 (1.02)/54.81(12.75)	4.26 (0.90)/56.99 (8.46)	<**0.001**/0.422
N3 (h)/(% of TST)	0.63 (0.51)/15.05 (12.53)	0.99 (0.57)/13.29 (7.81)	**0.010**/0.498
REM sleep (h)/(% of TST)	0.94 (0.50)/19.42 (10.12)	1.58 (0.75)/20.66 (8.36)	<**0.001**/0.611

*AUDIT* alcohol use disorders identification test, *ADS* alcohol dependence scale, *CIWA* the Clinical Institute withdrawal assessment for alcohol, *TLFB* the timeline follow-back for daily alcohol consumption in the last 90 days prior to the study, *LDH* the lifetime drinking history for the total lifetime alcohol consumption, *FTND* Fagerström test for nicotine dependence; *STAI* state-trait anxiety inventory, *BDI* the Beck depression inventory.

^a^One milligram of Diazepam was converted into 3 mg Oxazepam.

The bold values highlight that they are statistically significant.

Sleep (two nights) and MRI data (one MRI scan) of AUD participants were acquired between day 2–6 of their detoxification at the NIAAA inpatient unit in the NIH Clinical Center. Nicotine and caffeine use were allowed during patients’ inpatient stay. Nicotine and caffeine use were not correlated with sleep or sleep-related GM in AUD participants (see “Results” section). Benzodiazepines were given only if moderate or severe withdrawal symptoms emerged (Table 1). The use of benzodiazepines was controlled as a covariate in the sleep analyses because benzodiazepines can suppress SWS, although there is also evidence that SWA-generating mechanisms are not impaired by benzodiazepines [25–27]. The sleep assessments of HCs were also performed at the NIAAA inpatient unit after a night of adaptation. HCs were provided with caffeine-free beverages during their overnight stays and were scanned the following day. Both AUDs and HCs reported relatively low daily caffeine use, which was assessed on the day of admission to the unit and there were no group differences in caffeine consumption (Table 1). Care was taken to ensure that none of the participants ingested any alcohol while at the inpatient unit. They all had a negative urine drug screen and a negative breath test result for alcohol consumption on the days of testing. All participants provided written informed consent, which was in accordance with the declaration of Helsinki and was approved by the Institutional Review Board at the National Institutes of Health. Assuming *α* = 0.05 and *β* = 0.2, the current sample size allowed us to detect an effect size of 0.73.

### Sleep monitoring and stage scoring

For overnight sleep monitoring, we used an ambulatory device (the X4 Sleep Profiler, Advanced Brain Monitoring) that provides three channels of frontal EEG, a pulse rate, and a sensor to detect head movement [28]. This multichannel frontopolar EEG recording device yields an comparable sleep architecture estimates to full polysomnography (PSG) [29, 30]. Sleep stages were auto-scored using a web-based portal with an automated scoring algorithm [31, 32]. See Supplementary section “Sleep stage scoring and justification” for detailed information.

### MRI acquisition and preprocessing

Participants underwent MRI on a 3.0 T Magnetom Prisma scanner (Siemens Medical Solutions USA, Inc., Malvern, PA) equipped with a 32-channel head coil. T1-weighted 3D magnetization-prepared gradient-echo (MP-RAGE; TR/TE = 2400/2.24 ms, FA = 8 deg) and variable flip angle turbo spin-echo [33] (Siemens SPACE; TR/TE = 3200/564 ms) pulse sequences were used to acquire high-resolution anatomical brain images with 0.8 mm isotropic voxels field-of-view (FOV) = 240 × 256 mm, matrix = 300 × 320, and 208 sagittal slices.

### Cortical thickness

Structural images were first minimally preprocessed by the Human Connectome Project (HCP) standardized pipelines [34]. FreeSurfer version 5.3.0 (http://surfer.nmr.mgh.harvard.edu) was then used to compute the pial and white matter surfaces and to segment the anatomical MRI scans into 68 cortical parcels. We used the estimated average CT for each of the cortical parcels in the Desikan–Killiany atlas [35].

### Grey matter density

We assessed GMD using voxel-based morphometry (VBM) [36]. Structural images were first co-registered to the MNI average template distributed with SPM12 (Wellcome Trust Center for Neuroimaging; http://www.fil.ion.ucl.ac.uk/) using normalized mutual information. Bias-corrected structural images were created to reduce the influence of intensity inhomogeneity on segmentation. Structural images were then segmented into GM, white mater, cerebrospinal fluid, soft tissue, skull, and non-brain regions using unified segmentation [37]. The tissue class images created during segmentation were then used to generate a custom template using diffeomorphic anatomical registration through exponentiated lie algebra (DARTEL) and an 8 mm FWHM isotropic Gaussian kernel [38]. A binary mask was created from the mean of all participants’ smoothed DARTEL-normalized GM images, thresholded at 0.1 and used as an explicit mask in the statistical analyses of GM. To compare cortical GMD with CT, we wrapped the cortical volumetric data to the surface and estimated average GMD for each parcel in the Desikan–Killiany atlas [35].

### Statistical analyses

The whole-brain vertex/voxel-wise analyses were performed for CT and GMD. For CT, we used nonparametric permutation testing with FSL’S PALM software for multiple comparison correction with spatial statistics for each hemisphere separately (familywise error [FWE] *P*_FWE_ = 0.05, 10,000 permutation), and then Bonferroni-corrected for two hemispheres (*P*_FWE_ = 0.05/2). For GMD, clusters were corrected for multiple comparisons using cluster-level *P*_FWE_ < 0.05 with a cluster-defining threshold of *P* = 0.001, and cluster size >100 voxels. Two-sample *t*-tests were used to examine group differences in total sleep time (TST), REM, N2, N3, negative mood, CT, and GMD. A generalized linear regression model (GLM) was applied for the independent effects of group, sleep (REM, N2, and N3) and their interactions (i.e., to examine whether sleep-related CT/GMD differed between two groups). Next, we additionally added age and gender as covariates in the original GLM models to examine their potential effects. For CT, we also conducted ROI analyses that provides a rough overview of spatial distribution of CT variations and corrected for multiple comparison using the false discovery rate Benjamin−Hochberg method (FDR). The correlation between CT and cortical GMD was also examined. Finally, we tested whether total lifetime drinks (TLD) were associated with sleep and sleep-related CT/GMD in AUD. Age, gender, caffeine use, anxiety, depression, nicotine dependence, and benzodiazepine use were controlled, if they were correlated with group, TLD, CT/GMD, or sleep (N3 and REM). We reported our analyses both uncorrected and corrected for these potential covariates and explored the effect of age*alcohol on REM and REM-related CT/GMD. To examine the potential confounding effects of nicotine and caffeine use in AUD participant an independent *t*-test was performed to compare sleep stages and sleep stage related GM in AUD smokers (*n* = 19) vs. AUD non-smokers (*n* = 17). Additionally, Pearson’s correlation was used to examine the correlation between Fagerstrom Test for Nicotine Dependence (FTND) score, self-reported daily caffeine consumption and sleep and GM.

### Mediation analyses

We hypothesized that both CT and GMD would mediate the effect of chronic alcohol use on N3 and REM. To test this, we examined (1) whether group differences (categorical variable AUD = 1, HC = 0) in N3 and REM were mediated by GM structure, and (2) whether GM structure mediated the association between TLD and sleep (N3 and REM) in AUD. Mediation analyses (Fig. S1) were performed with the toolbox PROCESS v3.4 for SPSS 22 (IBM Corp., Armonk, NY) (bootstrap samples: 5000; confidence intervals: 95%) [39]. We ran the mediation analyses separately for CT and GMD and for N3 and REM.

## Results

### Group differences in sleep, negative mood, and GM structure

**Fig. 1:**
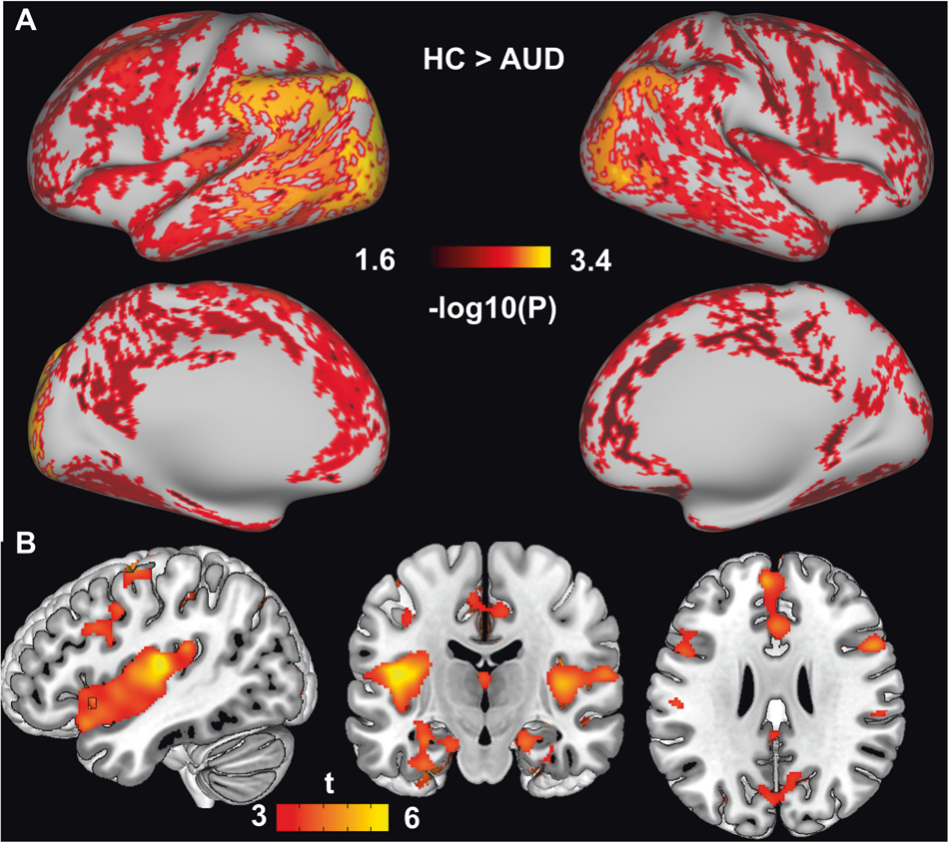
Group differences in GM structure (HC>AUD). Group differences in **A** CT: Color bar represents −log10(*p*) value. The significance threshold was −log10(0.05/2) = 1.602, Bonferroni-corrected for two hemispheres. and **B** GMD: Color bar represents *t* values.

AUD had shorter sleep duration in TST, REM, N2, and N3 and higher anxiety and depression ratings than HC (all *t*(60) < −2.67, *p* < 0.01) (Table 1). REM and N3 sleep were not associated with each other in either group (|*r*| < 0.258, *p* > 0.203). The whole-brain analyses (two-sample *t*-tests) revealed overall lower CT (*p* < 0.05/2) that was most prominent in the occipital cortex (Fig. 1A), and reduced GMD throughout frontal, occipital, parietal and temporal regions that was most prominent in the opercular cortex and insula (*p* < 0.001 with *k* > 100 and cluster-level *p*_FWE_ < 0.05) (Fig. 1B and Table 2).

**Table 2:** Group differences in gray matter density (two-sample *t*-test).

Brain areas	BA	MNI coordinates (*x* *y* *z*)	*K*	*T*
**HC>AUD**				
Central Opercular cortex	48	−45 −18 14	13,286	6.20
Insula	48	38 −9 12	9653	5.83
Precentral cortex_R	6	30 −3 52	2762	5.58
Precentral cortex_L	6	−38 −4 60	2098	5.46
Subcallosal cortex	25	0 14 −3	2852	5.39
Lingual	19	33 −81 −16	6722	5.23
Lateral occipital cortex (superior)	19	−39 −81 16	760	5.18
Superior frontal gyrus	32	−6 51 27	8560	5.08
Parahippocampal gyrus	36	26 −10 −33	1087	4.97
Temporal pole	28	24 8 −26	536	4.96
Lateral occipital cortex (inferior)	37	−45 −62 15	505	4.67

*BA* Brodmann area, *K* cluster size.

### Sleep-CT association

We included group (AUD vs. HC), duration of sleep stages (REM, N2 and N3) and their interactions in the GLM. For the combined group, whole-brain vertex-wise analysis showed longer N3 sleep was significantly associated with greater CT in the right insula and temporal gyrus (Fig. S2) and REM sleep was positively correlated with CT in the left hemisphere especially motor, temporal, parietal, and occipital cortices (Fig. S3A).

**Fig. 2:**
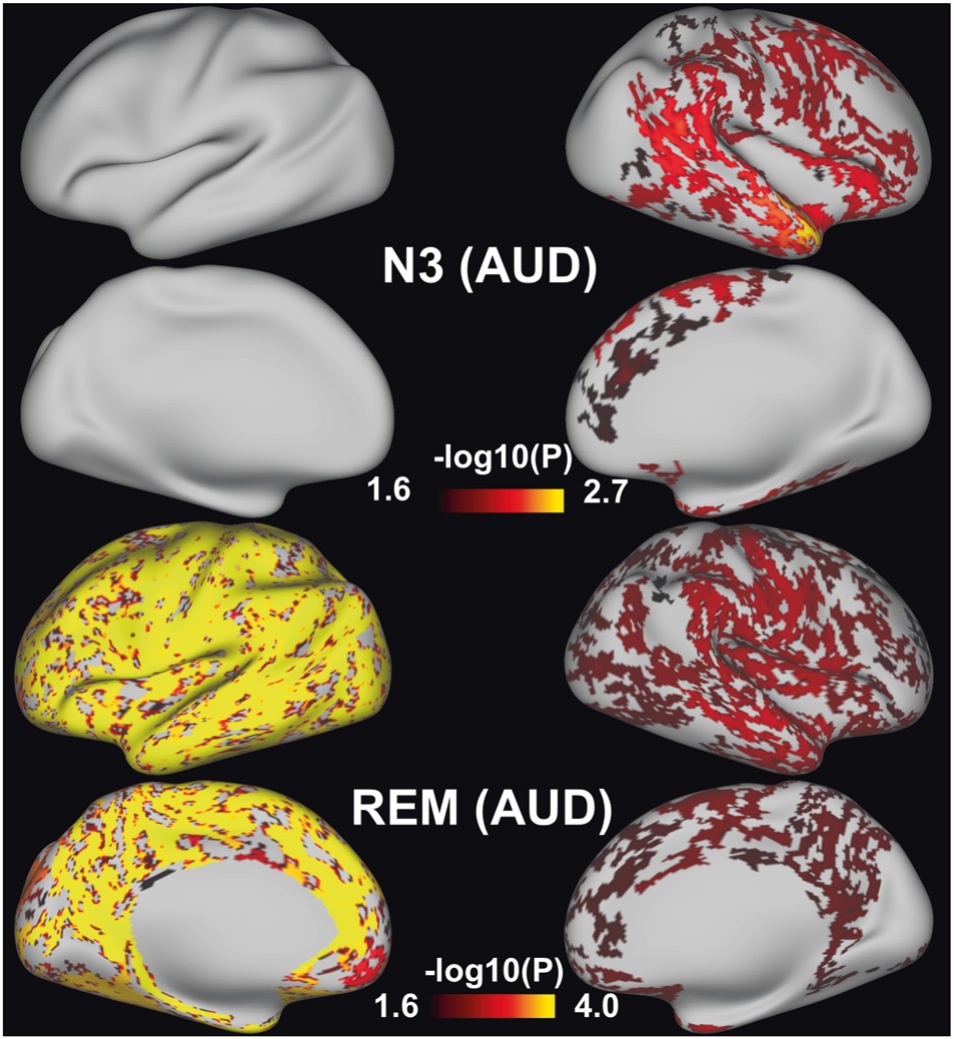
Sleep-CT association in AUD. N3-related and REM-related CT in AUD. The significance threshold was −log10(0.05/2) = 1.602, Bonferroni-corrected for two hemispheres.

**Fig. 3:**
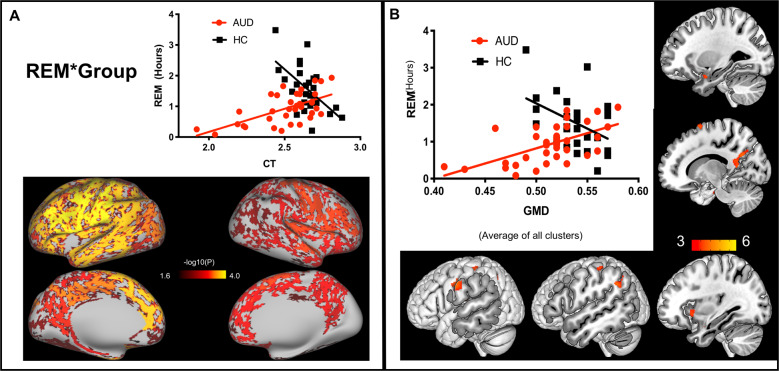
Effect of REM by group interaction on GM structure. REM sleep was differently correlated with GM in AUD vs. HC. **A** CT for the left motor, temporal, and parietal cortex. The significance threshold was −log10(0.05/2) = 1.602, Bonferroni-corrected for two hemispheres; **B** GMD for the left postcentral gyrus, supramarginal gyrus, amygdala, and Insula and right supplementary motor cortex and precuneus. Color bar represents *t* values. The correlation between REM time and CT/GMD (averaged across all the significant clusters) was plotted to demonstrate the direction of the interactions.

These combined results were driven by associations in AUD as revealed by the separate group analyses showing that in AUD only, N3 sleep was associated with CT in the right frontal (including anterior midline regions), insula, temporal, and parietal cortices and longer REM sleep was associated with CT throughout the whole brain, prominently in the left hemisphere (Fig. 2). In HC, the correlations were not significant after FWE correction. However, while not significant, the association in HC were opposite to that in AUD, such that longer REM was associated with less CT and the group differences in the REM-CT association were significant (Fig. 3A). The results remained after correcting for age and gender except that the association in AUD between N3 and CT was restricted to the left temporal pole (Fig. S4). Also see Supplementary section “Results of ROI analyses for CT: Table S1, Table S2, and Fig. S5” for ROI results. No N2-related CT correlations or group interactions were found.

### Sleep-GMD association

**Fig. 4:**
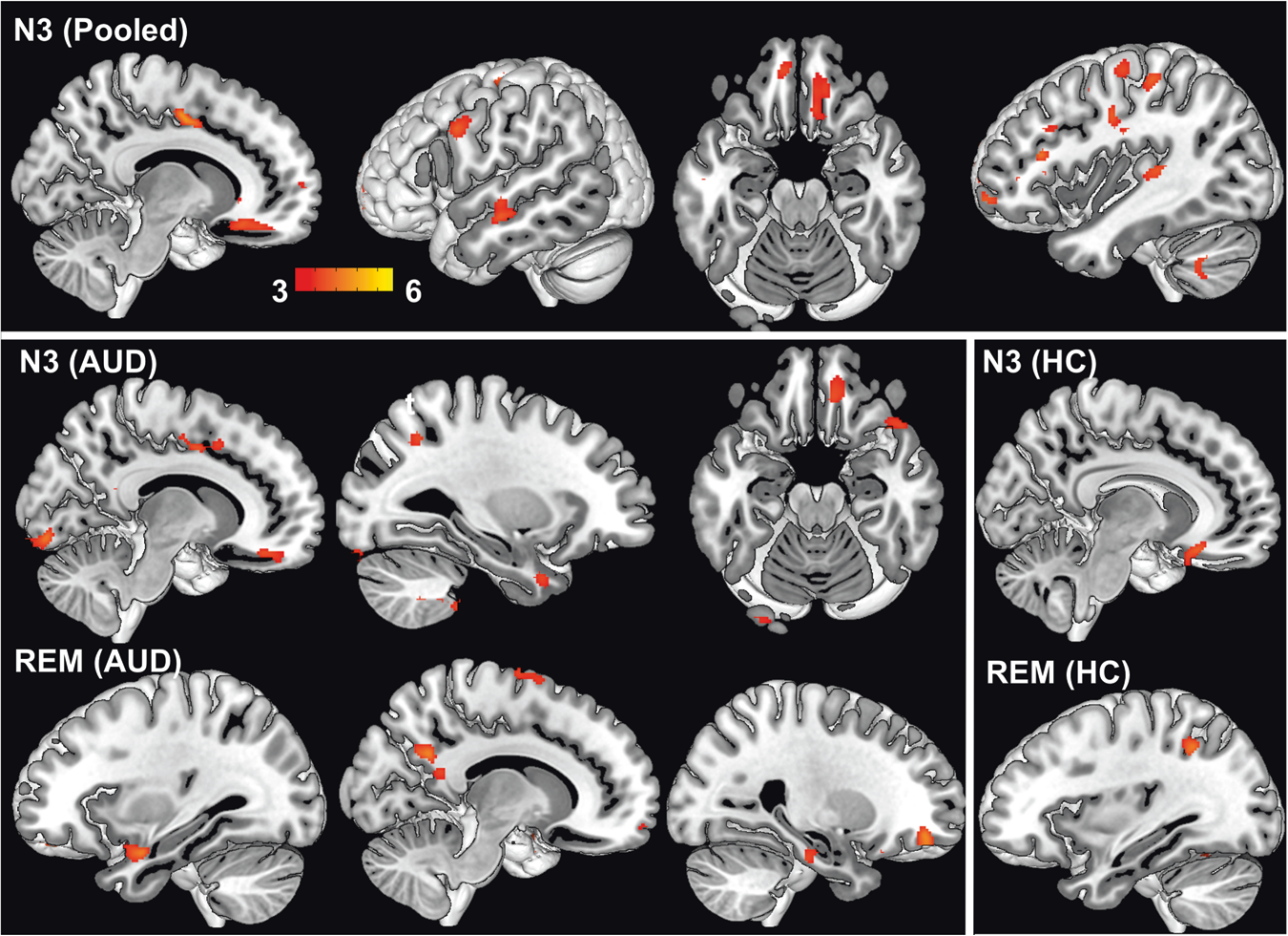
Sleep-GMD association. N3**-**related GMD in AUD and HC pooled together (upper panel). N3 and REM-related GMD in AUD only (lower panel left) and in HC only (lower panel right). Color bar represents *t* values.

For the combined group, whole-brain voxel-wise analyses showed that longer N3 sleep was associated with higher GMD in cerebellum, supplementary motor cortex, prefrontal cortex, and temporal gyrus (Table 3A and Fig. 4) and longer REM sleep was associated with higher GMD in cerebellum and left temporal pole (Table 3A and Fig. S3B). The correlation between N3 and GMD did not differ between groups. However, separate group analyses showed a larger range of regions with N3-GMD associations for AUD (right OFC, cerebellum, dorsal cingulate, and occipital cortex) than for HC (right OFC) (Table 3A and Fig. 4). Similar to the REM-CT association, we found an interaction between REM and group for GMD in motor cortex, precuneus, left supramarginal cortex, left amygdala and insula (Table 3A and Fig. 3B). AUD displayed positive correlations between REM and GMD in midline DMN, motor, and paralimbic regions, whereas HC showed negative correlations in the left supramarginal gyrus (Table 3A and Fig. 4). The results of N3-GMD association remained unaltered after including age and gender, while the interaction effect of REM-GMD for the separate groups and the REM*group interaction disappeared (Table 3B). With further explorative analyses, we found that GM structure mediated the effect of age on REM sleep in AUD (results below). In AUD but not in HC, cortical GMD was correlated with CT (Fig. S6). No N2-related GMD or interaction was found.

**Table 3:** A) Associations between sleep and gray matter density (GLM). B) Associations between sleep and gray matter density (GLM) (Controlled for age and gender).

A) Brain areas	BA	MNI coordinate (*x* *y* *z*)	*K*	*T*	Brain areas	BA	MNI coordinates (*x* *y* *z*)	*K*	*T*
**HC>AUD***(controlled for sleep*^a^*)*									
Precentral	6	−34 −6 45	237	4.29					
Middle temporal	21	−69 −26 −8	280	4.22					
**N3 (AUD&HC)***(controlled for group)*					**N3 (AUD)**				
Cerebellum8_R		38 −50 −45	1074	5.28	Cerebellum8_R		36 −50 −46	588	4.66
Cerebellum_Crus2_R		38 −81 −45	824	4.71	Cerebellum8_L		−32 −52 −46	238	4.03
Cerebellum8_L		−32 −51 −46	642	4.82	Paracingulate	32	15 12 42	382	4.18
Supplementary motor cortex	6	12 −6 45	223	5.15	Medial frontal	11	9 39 −20	226	3.98
Anterior cingulate	25	−8 26 3	1201	5.01	Inferior frontal	45	−46 38 0	212	3.94
Middle frontal	9	−32 16 48	200	4.88	Temporal pole	38	40 24 −22	238	4.03
Superior temporal	22	−68 −28 10	195	4.81	Lingual	18	14 −86 −9	648	4.79
Planum temporal	41	−38 −32 8	279	4.50	**N3** (HC)				
Middle temporal	21	−62 −15 −12	599	4.45	Orbitofrontal	11	9 26 −18	200	4.33
Central opercular	48	40 −3 22	334	4.45					
Insula	48	−38 −15 27	424	4.45					
**REM (AUD&HC)** (*controlled for group*)					**REM (AUD)**				
Cerebellum8_L		−24 −60 −45	193	4.61	Postcentral	3	−42 −34 54	279	4.78
Cerebellum8_R		28 −63 −46	209	4.28	Supplementary motor cortex	6	9 −3 74	230	4.23
Temporal pole	36	−28 8 −33	432	4.45	Parahippocampal_L	36	−32 −2 −24	560	4.63
REM*Group (AUD>HC)					Orbitofrontal	11	24 46 −14	309	4.81
Postcentral_L	3	−40 −33 54	336	4.86	Orbitofrontal	11	−20 50 −20	201	4.37
Postcentral_L	43	−64 −4 32	395	4.46	Precuneus	23	14 −56 28	370	4.91
Supplementary motor cortex	6	10 8 69	247	4.45	Parahippocampal_R	30	28 −21 −27	188	3.91
Precuneus	23	14 −60 27	507	4.33	**REM** (HC *negative association*)				
Supramarginal	40	−52 −51 34	270	4.33	Supramarginal	40	−32 −50 40	216	4.60
Amygdala		−28 0 −22	252	3.99					
Insula	48	−33 15 −6	178	3.89					

B) Brain areas	BA	MNI coordinates (*x* *y* *z*)	*K*	*T*	Brain areas	BA	MNI coordinates (*x* *y* *z*)	*K*	*T*
**N3 (AUD&HC)***(controlled for group)*					**N3 (AUD)**				
Cerebellum_8		39 −51 −44	934	5.13	Lingual	18	14 −86 −9	174	4.57
Cingulum_mid	6	12 −6 45	198	5.04	Cerebellum_8_R		38 −51 −46	797	4.79
Anterior cingulate	25	−10 26 −3	1017	4.79	Inferior frontal	45	−48 38 0	191	4.21
Cerebellum_8		−36 −52 −45	594	4.76	Cerebellum_8_L		−33 −54 −46	299	4.19
Cerebellum_Crus1_R		40 −81 −32	190	4.67	Inferior parietal	7	−27 −56 40	185	4.60
Cerebellum_Crus2		38 −81 −44	730	4.57	Cerebellum_Crus2		−16 −87 −46	274	4.17
Heschl’s Gyrus	41	−34 −33 6	183	4.24	Medial frontal	11	10 38 −18	194	3.83
Central opercular	48	42 −6 22	187	4.27	**N3 (HC)**				
Orbitofrontal _mid	47	−38 56 −8	218	4.21	Rectus	11	9 26 −18	177	4.35
Temporal_mid	21	−62 −15 −12	312	4.24	Cerebellum_9		4 −38 −54	184	4.28
**REM (AUD&HC)***(controlled for group)*									
Cerebellum_8_L		−22 −60 −45	212	4.27					
Cerebellum_8_R		28 −63 −46	275	4.49					
Fusiform	36	−27 8 −40	270	4.23					

^a^The observed group differences here are independent of sleep differences in AUD vs. HC (sleep measures as covariates were regressed out) and are likely contributed by other unknown factors.

### Covariates

We further controlled other covariates. The correlations between GM structure (averaged CT or GMD across the related regions) and sleep (N3 and REM) remained significant after correcting for age, anxiety, depression, and benzodiazepine use (all |*β*| > 0.664, all |*t*| > 3.60, all *p* < 0.001) (see Table 4 for covariates for GM structure and sleep). In HC but not in AUD, REM-related GMD was negatively correlated with anxiety and depression and more REM sleep was associated with higher anxiety scores (Table 4).

**Table 4:** Correlations between total lifetime drinks (TLD), gray matter structure (CT and GMD), sleep, and potential covariates in AUD and HC.

	TLD (AUD/HC)	N3-related GMD (AUD/HC)	REM-related GMD (AUD/HC)	N3-related CT (AUD)	REM-related CT (AUD)	N3 (AUD/HC)	REM (AUD/HC)
Age	0.303/0.544*	−0.481**/−0.462*	−0.586**/−0.529**	−0.481**	−0.507**	−0.133/−0.198	−0.335*/0.276
Gender	−0.163/−0.322	−0.180/0.033	−0.228/0.063	−0.191	−0.117	−0.181/0.377	−0.241/−0.116
Caffeine use	0.203/0.278	−0.041/−0.268	−0.019/−0.069	−0.047	0.009	−.135/-.296	0.206/0.125
STAI anxiety	0.465**/0.092	0.073/−0.192	0.077/−0.518**	0.106	0.157	−0.066/0.086	0.058/0.530**
BDI	0.186/0.143	0.132/−0.208	0.109/−0.442*	0.066	0.153	−0.074/0.069	0.066/0.388
FTND (AUD)	0.059	0.179	0.078	0.221	0.217	0.094	0.116
Benzo use (AUD)	0.531**	−0.430**	−0.353*	−0.471**	−0.304	−0.230	−0.215

*Correlation is significant at the 0.05 level (two-tailed).

**Correlation is significant at the 0.01 level (two-tailed).

The bold values highlight that they are statistically significant.

### TLD correlate with N3 and GM in AUD

In AUD, TLD were associated with shorter N3 sleep (*r*_s_(35) = −0.358, *p* = 0.035) but not with duration of other sleep stages or TST and this correlation was not altered after controlling for benzodiazepine use and anxiety (*β* = −0.481, *t* = −2.28, *p* = 0.030) (See Table 4 for covariates for TLD and N3). TLD were also negatively correlated with N3-related CT (*β* = −0.379, *t* = −2.64, *p* = 0.013) and GMD (*β* = −0.338, *t* = −2.25, *p* = 0.031), but not REM-related GM structure after regressing out age. Age was negatively associated with both CT and GMD (all *β* < −0.382, all *t* < −2.55, all *p* < 0.016). Of note, although benzodiazepine use was correlated with TLD and GM structure in AUD (Table 4), we did not include it as a covariate because it is unlikely that short-term benzodiazepine use affected GM structure and mediated the correlation between TLD and N3. Instead, AUD participants with greater TLD and GM reduction seemed to have required more benzodiazepine to control their withdrawal symptoms.

### GM structure mediates the effect of chronic alcohol use on N3 sleep

**Fig. 5:**
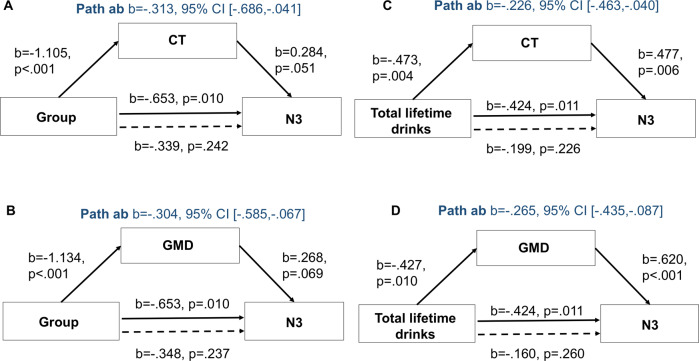
Mediation effects. **A** CT and **B** GMD mediated the group effect on N3 sleep. **C** CT and **D** GMD mediated the relationship between total lifetime drinks and N3 sleep in AUD.

The mediation analyses showed that group differences in N3 sleep were explained by group differences in GM (GMD: 46.55%, CT: 47.93% of total effect) (Fig. 5A, B). In AUD, the association between TLD and N3 sleep was significantly mediated by GMD (62.50% of total effect) and CT (53.30% of total effect) in N3-related regions (Fig. 5C, D).

### GM structure mediates the effect of age on REM sleep in AUD

We observed a negative association between age and REM in AUD but not in HC (Table 4). Age was negatively correlated with REM-related GM variations (Table 4). Thus, we conducted follow-up mediation analyses in AUD and revealed that the effect of age on REM was attributed to GM (both CT and GMD) in AUD (Total effect: *b* = −0.334, *p* = 0.046; CT: Indirect effect: *b* = −0.403, 95% CI [−0.761, −0.121]; direct effect: *b* = 0.069, *p* = 0.687; GMD: Indirect effect: *b* = −0.420, 95% CI [−0.768, −0.195]; direct effect: *b* = 0.086, *p* = 0.594).

As the effect of age on REM differed between groups (Table 4), we performed conditional process analyses (i.e., moderated mediation analyses) using GM as mediator and group as the moderator to further explore the age × alcohol interaction (Fig. S7). The moderated mediation effect was significant for both GMD and CT. GM mediated the age × alcohol/group interaction effect on REM sleep.

### Nicotine and caffeine effects

In the current study, half of AUD patients used nicotine, while none of the HC were smokers and caffeine and nicotine use were allowed during AUD detoxification. Thus, we further examine the effect of nicotine and caffeine use on sleep and sleep-related GM. To address potential effects from nicotine we compared AUD smokers versus AUD non-smokers and showed that they did not differ in TST, N3, REM, or sleep-stage related GMD and CT (all *t* < 1.61, all *p* > 0.117). Also, in AUD participants, sleep and sleep-related GM were not correlated with FTND scores or self-reported caffeine use.

## Discussion

In AUD, the prominent reductions in GM structure (both in CT and GMD) were associated with disrupted sleep (reduced duration of N3 and REM). For most brain regions (except medial prefrontal cortex) the patterns of associations with GM structure differed for N3 vs. REM and between HC vs. AUD. For CT, the associations with sleep, which were significant in AUD but not in HC, were lateralized such that CT reductions in right hemisphere were associated with shorter N3, whereas in left hemisphere were associated with shorter REM sleep. For GMD, a larger range of regions showed an association with N3 in AUD (right OFC, cerebellum, dorsal cingulate, and occipital cortex) than in HC (right OFC) and the association with REM revealed positive correlations in AUD with midline DMN, motor, and paralimbic regions, whereas negative correlations in HC with the left supramarginal gyrus. The mediation analyses revealed that changes in GM structure mainly mediated the effect of chronic alcohol use on the duration of N3 and an age by alcohol effect on REM sleep.

### GM reduction in AUD

Early postmortem studies reported focal neuronal loss in AUD throughout frontal, parietal, temporal, and occipital regions [40]. Subsequently brain imaging studies have consistently reported reductions in brain GM in patients with AUD compared to HC and even when compared to patients with other substance use disorders [12, 13, 15, 41]. Our results replicate these findings showing that AUD participants had lower CT [12, 15] and GMD [13, 41] in most brain regions including frontal lobe, insula, anterior and posterior cingulate cortex, motor areas (precentral and postcentral gyrus), temporal, parietal, and lateral occipital cortex.

### SWS and GM structure in AUD

The correlation between SWS and GM has been well-documented. From early childhood to late adolescence, the topographical distribution of SWA parallels GM development shifting from posterior to anterior regions [42, 43]. Furthermore, evidence supports the mediation effect of GM structure on the age-related SWA changes during adolescence and adulthood [18, 19, 44] and GM contributions to inter-individual differences in SWA among young adults [17].

As expected, the AUD participants who were studied during recent alcohol withdrawal had shorter duration of SWS than age-matched and gender-matched HC. SWA is dominant over prefrontal EEG derivation in adults and frequently initiated in frontal regions [44–46]. Consistent with previous studies [17, 44], we observed a significant association between SWS and GMD in OFC both in AUD and HC, particularly the right OFC. Our findings not only support the contribution of GMD variations in medial prefrontal cortex to inter-individual differences in SWS, but also provide evidence that GMD changes in this region underlie the reduced SWS in AUD beyond that from age effects. Consistently, SWS-CT correlations in right frontal regions especially OFC and anterior midline regions was observed in AUD. However, unlike GMD, no SWS-related association with CT was observed in HC. As shown in Fig. S6, the lack of a correlation between GMD and CT in HC might account for these differences, which stresses the necessity to assess both of these GM measures as they might offer complementary information about GM structure [23].

GM structure (both GMD and CT) was associated with SWS in a larger extent of regions in AUD than HC including cerebellum, frontal, cingulate, temporal and lingual cortex. SWS-related GM variations in these regions have been reported in adults [17, 18]. While multiple frontal, cingulate and temporal regions are assumed to relate to SW source generation [6], mounting evidence also shows the involvement of posterior brain regions and cerebellum in SWS [5, 17, 18, 47, 48]. This might reflect the global cortical synchrony and cortico-cerebellar interactions during SWS [7, 49, 50]. In the current study, the GM deficits in AUD including those in medial prefrontal cortex that are relevant for SWS-initiation might shift the responsibility for generating and maintaining SWS to other regions [44–46]. Similarly, poor sleep quality was associated with CT for a broader set of brain regions in patients with insomnia than in HC [51]. The engagement of more expanded areas of activation while performing specific tasks is frequently observed as a compensation mechanism in patients with brain lesions [52, 53]. Further mediation analyses revealed that changes in GM structure not only explained group differences in SWS, but also mediated the association between TLD and SWS in AUD.

### REM and GM structure in AUD

In the current study, the correlation between REM and GM structure differed between AUD and HC. While AUD participants showed strong positive correlations between REM and GM in OFC, motor (pre-central and postcentral, supplementary motor cortex), limbic (amygdala, insula, parahippocampal gyrus), temporal and parietal (precuneus, left inferior parietal cortex) regions, HC displayed negative correlations in these regions, particularly in the left inferior parietal cortex. Early ^18^FDG PET studies reported elevated brain glucose metabolism in the left amygdala, insula, parahippocampal gyri, and midline frontal regions including OFC, ACC, and medial prefrontal cortex and reduced metabolism in left inferior parietal cortex, precuneus, and posterior cingulate cortex during REM sleep compared to wakefulness [9–11]. Furthermore, patients with RBD showed GM reduction in regions that overlapped with our findings on REM-related regions in AUD [20–22]. Supporting previous findings [5, 6, 9, 11], we found GM reductions in anterior midline regions associated with both SWS and REM sleep.

The negative correlation between REM and GMD in the left inferior parietal regions in HC was unexpected. The opposite findings in AUD vs. HC suggested an inverted U-shaped relationship between GMD and REM, which might relate to the deactivation of this region during REM sleep [9]. REM-related GMD was negatively correlated with anxiety and depression and more REM sleep was associated with higher level of negative mood in HC but not in AUD (Table 4). Similar observations have been reported in major depressive disorder [54, 55].

The age by alcohol/group effect on REM sleep was also unexpected. A large population-based study reported decreased REM and SWS with increased age [56]. It has also been reported that REM sleep mediates age-related decline in prospective memory consolidation [57]. In the current study, the absence of age-related decline in REM and SWS in HC likely reflects our sample size and smaller age range (22–63 years) than the other two studies (18/20–80 years). Accumulating evidence shows that alcohol accelerates brain aging as reflected by GM changes with the strongest effects in limbic, temporal, and frontal regions [13, 14, 58]. In line with this, our findings demonstrated that the correlation between age and CT was moderated by alcohol/group (Fig. S7). Furthermore, older adults appear to be more vulnerable to alcohol-related brain aging [58]. In our study, chronic alcohol might have accelerated the aging process in GM especially in the older AUD participants and GM changes then drove age-related decrease in REM sleep. Although the age by alcohol effect on REM sleep needs to be confirmed in a larger sample size, our results highlight the relevance of addressing alcohol use problems in older adults, which are frequently neglected clinically [59, 60]. Comorbid depression, anxiety, and AUD are common in older adults [61] and drinking increases the risk for late-life suicide [62]. As REM modulates emotional experiences and their regulation [63, 64], alcohol use in older adults might impede emotional processing to a greater extent than in younger individuals with AUD increasing their risk for severe adverse outcomes.

### Lateralization of sleep-GM association

In general, we observed a N3-GM association in the right hemisphere and REM-GM association in the left hemisphere, especially for CT. The right lateralization of SWS has been reported previously. In HC, slow waves originate more often in the right relative to the left hemisphere [65]. Delta counts in the right frontal and central regions were significantly greater than those of the left during all-night sleep [66] and the rightward asymmetry increases along with increased sleep depth [67]. Furthermore, callosotomy enhances N3-related asymmetry [65]. Thus, the white matter microstructure disruption in the corpus callosum reported in AUD might accentuate N3-related lateralization [65, 68, 69]. In contrast, REM lateralization is less supported by the literature and previous findings in HC are inconsistent [70, 71]. So, it unclear whether/how chronic alcohol use changes REM-related asymmetry and whether there is an age–alcohol interaction in this asymmetry [72].

### Clinical implication and future directions

In this study, we showed that GM changes in AUD are associated with sleep disturbances using ambulatory polysomnography. These findings are clinically relevant, because they provide preliminary evidence that sleep might serve as a biomarker of structural changes and might be useful to monitor recovery during AUD treatment. The wireless sleep device is amenable to a home-based environment, which facilitates its use. Furthermore, we identified a “hotspot” i.e., anterior DMN that was associated with both SWS and REM sleep in AUD as well as regions uniquely associated with different sleep stages. Treatments that target these regions with transcranial magnetic stimulation (TMS) or transcranial direct current stimulation (TDCS) might improve sleep and subsequently bring sleep-related cognitive and emotional benefits to AUD recovery [64, 73]. Though we interpret our findings to indicate that GM changes in AUD underlie the sleep disruption it is likely that the relationship between GM and sleep is bidirectional. Thus, treatments that improve SWS and REM might help restore structural brain changes including those in anterior DMN, which is sensitive to sleep [74, 75], and is affected in AUD as well as other substance use disorders [76].

## Limitations

The mediation analyses applied in this study measure associations and do not prove causality. Brain structural, functional, and neurochemical changes from alcohol use can cause sleep problems in AUD [77] and the induced sleep loss might sequentially lead to GM reduction [2, 78, 79]. Thus, it’s highly likely that the link between GM and sleep disturbance in AUD is bidirectional but the cross-sectional nature of our study cannot clarify the directionality of these associations. Longitudinal studies are needed to examine whether GM reduction precedes sleep disturbances in a larger population and to establish causality. Another limitation was the differences in nicotine consumption between the groups (half of AUD participants smoked whereas none of the controls did) since nicotine can alter sleep architecture, suppress N3, and REM sleep [80–83] and the co-use of alcohol and nicotine can worsen GM reduction [84]. However, it is not likely that our findings were significantly affected by nicotine use in AUD because FTND score was not correlated with N3, REM, or sleep-stage related GM (Table 4) and AUD smokers did not differ from AUD non-smokers in sleep or GM. However, our findings need to be replicated in future studies with larger sample sizes. Finally, though we excluded participants with mood disorders including major depressive disorder, AUD patients reported more depressive and anxious symptoms (Table 1) than HC and symptoms of anxiety and depression are known to contribute to sleep disruption [85]. On the other hand, negative mood is a key withdrawal symptom in AUD and one that is associated with AUD severity [86]. Thus, in our study, it is not possible to disentangle the extent to which sleep impairments in AUD contribute to negative mood or that negative mood during withdrawal contributes to sleep impairment.

## Conclusion

Together, our findings provide evidence for the association between GM atrophy and sleep disturbances in AUD. Strategies to ameliorate brain structural changes in AUD could benefit sleep and vice versa.

## Supplementary information

Supplemental material

## References

[1] Brower KJ, Perron BE (2010). Prevalence and correlates of withdrawal-related insomnia among adults with alcohol dependence: results from a national survey. Am J Addict.

[2] Koob GF, Colrain IM. Alcohol use disorder and sleep disturbances: a feed-forward allostatic framework. *Neuropsychopharmacology* 2019. 10.1038/s41386-019-0446-0. 10.1038/s41386-019-0446-0 PMC6879503 31234199

[3] Brower KJ, Aldrich MS, Hall JM (1998). Polysomnographic and subjective sleep predictors of alcoholic relapse. Alcohol Clin Exp Res.

[4] Feige B, Scaal S, Hornyak M, Gann H, Riemann D (2007). Sleep electroencephalographic spectral power after withdrawal from alcohol in alcohol-dependent patients. Alcohol Clin Exp Res.

[5] Dang-Vu TT, Schabus M, Desseilles M, Albouy G, Boly M, Darsaud A (2008). Spontaneous neural activity during human slow wave sleep. PNAS.

[6] Murphy M, Riedner BA, Huber R, Massimini M, Ferrarelli F, Tononi G (2009). Source modeling sleep slow waves. Proc Natl Acad Sci USA.

[7] Nir Y, Staba RJ, Andrillon T, Vyazovskiy VV, Cirelli C, Fried I (2011). Regional slow waves and spindles in human sleep. Neuron.

[8] Madsen PL, Holm S, Vorstrup S, Friberg L, Lassen NA, Wildschiødtz G (1991). Human regional cerebral blood-flow during rapid-eye-movement sleep. J Cereb Blood Flow Metab.

[9] Chong-Hwa Hong C, Gillin JC, Dow BM, Wu J, Buchsbaum MS (1995). Localized and lateralized cerebral glucose metabolism associated with eye movements during REM sleep and wakefulness: a positron emission tomography (PET) Study. Sleep.

[10] Maquet P, Péters J, Aerts J, Delfiore G, Degueldre C, Luxen A (1996). Functional neuroanatomy of human rapid-eye-movement sleep and dreaming. Nature.

[11] Nofzinger EA, Mintun MA, Wiseman M, Kupfer DJ, Moore RY (1997). Forebrain activation in REM sleep: an FDG PET study. Brain Res.

[12] Mackey S, Allgaier N, Chaarani B, Spechler P, Orr C, Bunn J (2019). Mega-analysis of gray matter volume in substance dependence: general and substance-specific regional effects. Am J Psychiatry.

[13] Thayer RE, Hagerty SL, Sabbineni A, Claus ED, Hutchison KE, Weiland BJ (2016). Negative and interactive effects of sex, aging, and alcohol abuse on gray matter morphometry. Hum Brain Mapp.

[14] Sullivan EV, Zahr NM, Sassoon SA, Thompson WK, Kwon D, Pohl KM (2018). The role of aging, drug dependence, and hepatitis C comorbidity in alcoholism cortical compromise. JAMA Psychiatry.

[15] Tomasi DG, Wiers CE, Shokri-Kojori E, Zehra A, Ramirez V, Freeman C (2019). Association between reduced brain glucose metabolism and cortical thickness in alcoholics: evidence of neurotoxicity. Int J Neuropsychopharmacol.

[16] Wiers CE, Gawron CK, Gröpper S, Spengler S, Stuke H, Lindenmeyer J (2015). Decreased gray matter volume in inferior frontal gyrus is related to stop-signal task performance in alcohol-dependent patients. Psychiatry Res.

[17] Saletin JM, van der Helm E, Walker MP (2013). Structural brain correlates of human sleep oscillations. Neuroimage.

[18] Dubé J, Lafortune M, Bedetti C, Bouchard M, Gagnon JF, Doyon J (2015). Cortical thinning explains changes in sleep slow waves during adulthood. J Neurosci.

[19] Goldstone A, Willoughby AR, de Zambotti M, Franzen PL, Kwon D, Pohl KM (2018). The mediating role of cortical thickness and gray matter volume on sleep slow-wave activity during adolescence. Brain Struct Funct.

[20] Rahayel S, Montplaisir J, Monchi O, Bedetti C, Postuma RB, Brambati S (2015). Patterns of cortical thinning in idiopathic rapid eye movement sleep behavior disorder. Mov Disord.

[21] Rahayel S, Postuma RB, Montplaisir J, Bedetti C, Brambati S, Carrier J (2018). Abnormal gray matter shape, thickness, and volume in the motor cortico-subcortical loop in idiopathic rapid eye movement sleep behavior disorder: association with clinical and motor features. Cereb Cortex.

[22] Campabadal A, Segura B, Junque C, Serradell M, Abos A, Uribe C (2019). Cortical gray matter and hippocampal atrophy in idiopathic rapid eye movement sleep behavior disorder. Front Neurol.

[23] Gennatas ED, Avants BB, Wolf DH, Satterthwaite TD, Ruparel K, Ciric R (2017). Age-related effects and sex differences in gray matter density, volume, mass, and cortical thickness from childhood to young adulthood. J Neurosci.

[24] Pomares FB et al. Beyond sleepy: structural and functional changes of the default-mode network in idiopathic hypersomnia. Sleep. 2019. 10.1093/sleep/zsz156. 10.1093/sleep/zsz156 PMC6802570 31328786

[25] Tobler I, Kopp C, Deboer T, Rudolph U (2001). Diazepam-induced changes in sleep: role of the alpha 1 GABA(A) receptor subtype. Proc Natl Acad Sci USA.

[26] Lehmann W. The effect of oxazepam on sleep in normal human volunteers. Acta Psychiatr Scand Suppl. 1978;58:33–9. 10.1111/j.1600-0447.1978.tb02385.x 216235

[27] Dijk D-J (2010). Slow-wave sleep deficiency and enhancement: implications for insomnia and its management. World J Biol Psychiatry.

[28] Levendowski DJ, Ferini-Strambi L, Gamaldo C, Cetel M, Rosenberg R, Westbrook PR (2017). The accuracy, night-to-night variability, and stability of frontopolar sleep electroencephalography biomarkers. J Clin Sleep Med.

[29] Finan PH, Richards JM, Gamaldo CE, Han D, Leoutsakos JM, Salas R (2016). Validation of a wireless, self-application, ambulatory electroencephalographic sleep monitoring device in healthy volunteers. J Clin Sleep Med.

[30] Lucey BP, Mcleland JS, Toedebusch CD, Boyd J, Morris JC, Landsness EC (2016). Comparison of a single-channel EEG sleep study to polysomnography. J Sleep Res.

[31] Stepnowsky C, Levendowski D, Popovic D, Ayappa I, Rapoport DM (2013). Scoring accuracy of automated sleep staging from a bipolar electroocular recording compared to manual scoring by multiple raters. Sleep Med.

[32] Popovic D, Khoo M, Westbrook P (2014). Automatic scoring of sleep stages and cortical arousals using two electrodes on the forehead: validation in healthy adults. J Sleep Res.

[33] Mugler JP, Bao S, Mulkern RV, Guttmann CR, Robertson RL, Jolesz FA (2000). Optimized single-slab three-dimensional spin-echo MR imaging of the brain. Radiology.

[34] Glasser MF, Sotiropoulos SN, Wilson JA, Coalson TS, Fischl B, Andersson JL (2013). The minimal preprocessing pipelines for the Human Connectome Project. NeuroImage.

[35] Desikan RS, Ségonne F, Fischl B, Quinn BT, Dickerson BC, Blacker D (2006). An automated labeling system for subdividing the human cerebral cortex on MRI scans into gyral based regions of interest. NeuroImage.

[36] Hutton C, Draganski B, Ashburner J, Weiskopf N (2009). A comparison between voxel-based cortical thickness and voxel-based morphometry in normal aging. Neuroimage.

[37] Ashburner J, Friston KJ (2005). Unified segmentation. Neuroimage.

[38] Ashburner J (2007). A fast diffeomorphic image registration algorithm. Neuroimage.

[39] Hayes AF. Introduction to mediation, moderation, and conditional process analysis, 2nd ed: a regression-based approach. New York: Guilford Publications; 2017.

[40] Courville CB. Effects of alcohol on the nervous system of man. Oxford: San Lucas Press; 1955.

[41] Makris N, Oscar-Berman M, Jaffin SK, Hodge SM, Kennedy DN, Caviness VS (2008). Decreased volume of the brain reward system in alcoholism. Biol Psychiatry.

[42] Shaw P, Kabani NJ, Lerch JP, Eckstrand K, Lenroot R, Gogtay N (2008). Neurodevelopmental trajectories of the human cerebral cortex. J. Neurosci.

[43] Kurth S, Ringli M, Geiger A, LeBourgeois M, Jenni OG, Huber R (2010). Mapping of cortical activity in the first two decades of life: a high-density sleep electroencephalogram study. J Neurosci.

[44] Mander BA, Rao V, Lu B, Saletin JM, Lindquist JR, Ancoli-Israel S (2013). Prefrontal atrophy, disrupted NREM slow waves and impaired hippocampal-dependent memory in aging. Nat Neurosci.

[45] Zeitlhofer J, Anderer P, Obergottsberger S, Schimicek P, Lurger S, Marschnigg E (1993). Topographic mapping of EEG during sleep. Brain Topogr.

[46] Massimini M, Huber R, Ferrarelli F, Hill S, Tononi G (2004). The sleep slow oscillation as a traveling wave. J Neurosci.

[47] Hofle N, Paus T, Reutens D, Fiset P, Gotman J, Evans AC (1997). Regional cerebral blood flow changes as a function of delta and spindle activity during slow wave sleep in humans. J Neurosci.

[48] Maquet P (2000). Functional neuroimaging of normal human sleep by positron emission tomography. J Sleep Res.

[49] Rowland NC, Goldberg JA, Jaeger D (2010). Cortico-cerebellar coherence and causal connectivity during slow-wave activity. Neuroscience.

[50] Canto CB, Onuki Y, Bruinsma B, van der Werf YD, De Zeeuw CI (2017). The sleeping cerebellum. Trends Neurosci.

[51] Suh S, Kim H, Dang-Vu TT, Joo E, Shin C (2016). Cortical thinning and altered cortico-cortical structural covariance of the default mode network in patients with persistent insomnia symptoms. Sleep.

[52] Heiss WD, Kessler J, Thiel A, Ghaemi M, Karbe H (1999). Differential capacity of left and right hemispheric areas for compensation of poststroke aphasia. Ann Neurol.

[53] Blasi V, Young AC, Tansy AP, Petersen SE, Snyder AZ, Corbetta M (2002). Word retrieval learning modulates right frontal cortex in patients with left frontal damage. Neuron.

[54] Singh MK, Kesler SR, Hadi Hosseini SM, Kelley RG, Amatya D, Hamilton JP (2013). Anomalous gray matter structural networks in major depressive disorder. Biol Psychiatry.

[55] Qiu L, Lui S, Kuang W, Huang X, Li J, Li J (2014). Regional increases of cortical thickness in untreated, first-episode major depressive disorder. Transl Psychiatr.

[56] Moraes W, Piovezan R, Poyares D, Bittencourt LR, Santos-Silva R, Tufik S (2014). Effects of aging on sleep structure throughout adulthood: a population-based study. Sleep Med.

[57] Scullin MK et al. Rapid eye movement sleep mediates age-related decline in prospective memory consolidation. Sleep. 2019. 10.1093/sleep/zsz055. 10.1093/sleep/zsz055 PMC6559169 30860593

[58] Guggenmos M, Schmack K, Sekutowicz M, Garbusow M, Sebold M, Sommer C (2017). Quantitative neurobiological evidence for accelerated brain aging in alcohol dependence. Transl Psychiatry.

[59] Sorocco KH, Ferrell SW (2006). Alcohol use among older adults. J Gen Psychol.

[60] Kuerbis A, Sacco P, Blazer DG, Moore AA (2014). Substance abuse among older adults. Clin Geriatr Med.

[61] Devanand DP (2002). Comorbid psychiatric disorders in late life depression. Biol Psychiatry.

[62] Blow FC, Brockmann LM, Barry KL (2004). Role of alcohol in late-life suicide. Alcoholism.

[63] van der Helm E, Yao J, Dutt S, Rao V, Saletin JM, Walker MP (2011). REM sleep depotentiates amygdala activity to previous emotional experiences. Curr Biol.

[64] Wassing R, Lakbila-Kamal O, Ramautar JR, Stoffers D, Schalkwijk F, Van Someren E (2019). Restless REM sleep impedes overnight amygdala adaptation. Curr Biol.

[65] Avvenuti G et al. Integrity of corpus callosum is essential for the cross-hemispheric propagation of sleep slow waves: a high-density EEG study in split-brain patients. J Neurosci. 2020. 10.1523/JNEUROSCI.2571-19.2020. 10.1523/JNEUROSCI.2571-19.2020 PMC7363462 32541070

[66] Sekimoto M, Kato M, Kajimura N, Watanabe T, Takahashi K, Okuma T (2000). Asymmetric interhemispheric delta waves during all-night sleep in humans. Clin Neurophysiol.

[67] McAvoy M, Mitra A, Tagliazucchi E, Laufs H, Raichle ME (2017). Mapping visual dominance in human sleep. NeuroImage.

[68] De Santis S, Bach P, Pérez-Cervera L, Cosa-Linan A, Weil G, Vollstädt-Klein S (2019). Microstructural white matter alterations in men with alcohol use disorder and rats with excessive alcohol consumption during early abstinence. JAMA Psychiatry.

[69] Pfefferbaum A, Rosenbloom MJ, Chu W, Sassoon SA, Rohlfing T, Pohl KM (2014). White matter microstructural recovery with abstinence and decline with relapse in alcohol dependence interacts with normal ageing: a controlled longitudinal DTI study. Lancet Psychiatry.

[70] Bolduc C, Daoust AM, Limoges T, Braun CMJ, Godbout R (2003). Hemispheric lateralization of the EEG during wakefulness and REM sleep in young healthy adults. Brain Cogn.

[71] Roth C, Achermann P, Borbely AA (1999). Frequency and state specific hemispheric asymmetries in the human sleep EEG. Neurosci Lett.

[72] Vyazovskiy VV, Borbely AA, Tobler I (2002). Interhemispheric sleep EEG asymmetry in the rat is enhanced by sleep deprivation. J Neurophysiol.

[73] Diekelmann S, Born J (2010). The memory function of sleep. Nat Rev Neurosci.

[74] Zhang R et al. Sleep inconsistency between weekends and weekdays is associated with changes in brain function during task and rest. Sleep 2020. 10.1093/sleep/zsaa076. 10.1093/sleep/zsaa076 PMC7551301 32333599

[75] Gujar N, Yoo S-S, Hu P, Walker MP (2010). The unrested resting brain: sleep deprivation alters activity within the default-mode network. J Cogn Neurosci.

[76] Zhang R, Volkow ND (2019). Brain default-mode network dysfunction in addiction. NeuroImage.

[77] Colrain IM, Nicholas CL, Baker FC (2014). Alcohol and the sleeping brain. Handb Clin Neurol.

[78] Liu C, Kong X-Z, Liu X, Zhou R, Wu B (2014). Long-term total sleep deprivation reduces thalamic gray matter volume in healthy men. Neuroreport.

[79] Joo EY, Tae WS, Lee MJ, Kang JW, Park HS, Lee JY (2010). Reduced brain gray matter concentration in patients with obstructive sleep apnea syndrome. Sleep.

[80] Jaehne A, Loessl B, Bárkai Z, Riemann D, Hornyak M (2009). Effects of nicotine on sleep during consumption, withdrawal and replacement therapy. Sleep Med Rev.

[81] Gillin JC, Lardon M, Ruiz C, Golshan S, Salin-Pascual R (1994). Dose-dependent effects of transdermal nicotine on early morning awakening and rapid eye movement sleep time in nonsmoking normal volunteers. J Clin Psychopharmacol.

[82] Davila DG, Hurt RD, Offord KP, Harris CD, Shepard JW (1994). Acute effects of transdermal nicotine on sleep architecture, snoring, and sleep-disordered breathing in nonsmokers. Am J Respir Crit Care Med.

[83] Zhang L, Samet J, Caffo B, Bankman I, Punjabi NM (2008). Power spectral analysis of EEG activity during sleep in cigarette smokers. Chest.

[84] Elbejjani M, Auer R, Jacobs DR, Haight T, Davatzikos C, Goff DC (2019). Cigarette smoking and gray matter brain volumes in middle age adults: the CARDIA Brain MRI sub-study. Transl Psychiatry.

[85] Sweetman A, Lovato N, Micic G, Scott H, Bickley K, Haycock J (2020). Do symptoms of depression, anxiety or stress impair the effectiveness of cognitive behavioural therapy for insomnia? A chart-review of 455 patients with chronic insomnia. Sleep Med.

[86] Koob GF, Volkow ND (2016). Neurobiology of addiction: a neurocircuitry analysis. Lancet Psychiatry.

