# Supplementary material for: Sleep disturbances are associated with cortical and subcortical atrophy in alcohol use disorder
Source: Transl Psychiatry. 2021 Aug 16;11:428. doi: 10.1038/s41398-021-01534-0 (PMC8368207; doi:10.1038/s41398-021-01534-0)
Supplement: Supplementary file 1 — Supplemental material [file 41398_2021_1534_MOESM1_ESM.pdf]

Sleep disturbances are associated with cortical and subcortical atrophy in alcohol use disorder
Supporting information

Participants: inclusion and exclusion criteria

Sleep stage scoring and justification

Figure S1 Mediation analyses

Figure S2 N3-CT association in AUD and HC pooled together

Figure S3 REM-GM association in AUD and HC pooled together

Figure S4 Significant sleep-CT associations after controlling for age and gender

Results of ROI analyses for CT

- ***Table S1 Group differences in CT (HC>AUD)***
- ***Table S2 Associations between sleep and CT in 68 parcels (FDR-corrected P<.05)***
- ***Figure S5 Sleep-CT associations (ROI analyses)***

Correlation between two grey matter measures

- ***Figure S6 Correlation between CT and GMD***

GM structure mediates the age*group interaction effect on REM sleep

- ***Figure S7 Modelling***
- ***Methods***
- ***Results***

References

Participants: inclusion and exclusion criteria

All AUD participants received a DSM 5 diagnosis of moderate or severe AUD *(1)* and had a minimum 5-year history of heavy drinking based on SAMSHA's criteria (i.e. for men, 5 or more drinks/day on at least 5 different days per month; and for women, 4 or more drinks/day on at least 5 different days per month). HCs were screened to exclude individuals with insomnia, restless leg syndrome, narcolepsy, obstructive sleep apnea and/or abnormal sleeping patterns including sleeping during the day, using medication to fall asleep, sleeping less than 5 hours per night or working in night shifts as determined by self-report and/or medical history. All participants had no major medical, neurological or psychiatric disorders (other than alcohol and nicotine use disorder in the AUD group or current tobacco smoking in either group). Participants with current DSM-IV or DSM 5 diagnose of a major psychiatric disorder (including depression and anxiety disorders) that required hospitalization or daily medications for over 4 weeks in the past year (i.e., antidepressants; anticholinergics; antipsychotics; anxiolytics; lithium; sedative/hypnotics, psychotropic drugs not otherwise specified including herbal products) and chronic benzodiazepine use prior to alcohol detox were excluded.

Sleep stage scoring and justification

Filtered and unfiltered power spectral data from the three frontopolar EEG channels were used to characterize the sleep stage as described in *(2)*. Of note, slow delta waves with amplitudes that exceeded ± 30 μV were used to identify N3 sleep by autoscoring *(2)* rather than ± 37.5 μV from American Academy of Sleep Medicine (AASM) guidelines *(3)*. The sleep profiler autoscoring has high agreement in identifying total sleep time, REM sleep, N2 and N3 sleep against full PSG staging by human scorers *(2,4)*. Because of the lack of occipital electrodes, the agreement of N1 sleep against full PSG is poor (2,4,5). Since we focused here on N3 and REM sleep, this shortage did not impede our ability to test our hypotheses. Furthermore, averaging the 2-night sleep measures we obtained for AUD patient improved the reliability of the sleep stage assessments *(2)*.

Figure S1 Mediation analyses

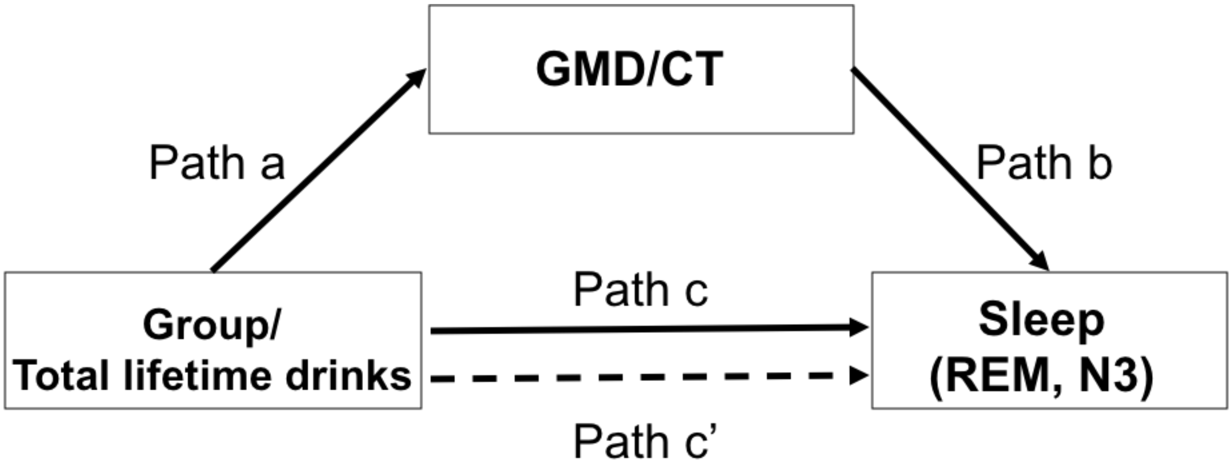

We calculated 1) the total effect of predictor group/total lifetime drinks on N3 and REM (Path c, i.e. equal to Path ab + Path c’), and 2) the direct effect of group/total lifetime drinks on N3 and REM in the presence of the mediator GM structure (Path c’), and 3) the effect of group/total lifetime drinks on N3 and REM via GM structure (the indirect effect Path ab).

We hypothesized that chronic alcohol use (AUD group membership and total lifetime drinks in AUD) exerts its total effect on sleep (N3 and REM) (path c) by an initial effect on GM structure (path a), which then drives reductions in sleep (path b). Path c’ (direct effect) indicates the effect of alcohol use on sleep independently of GMD/CT.

Figure S2 N3-CT association in AUD and HC pooled together

The significance threshold was -log10(0.05/2) =1.602, Bonferroni-corrected for two hemispheres.

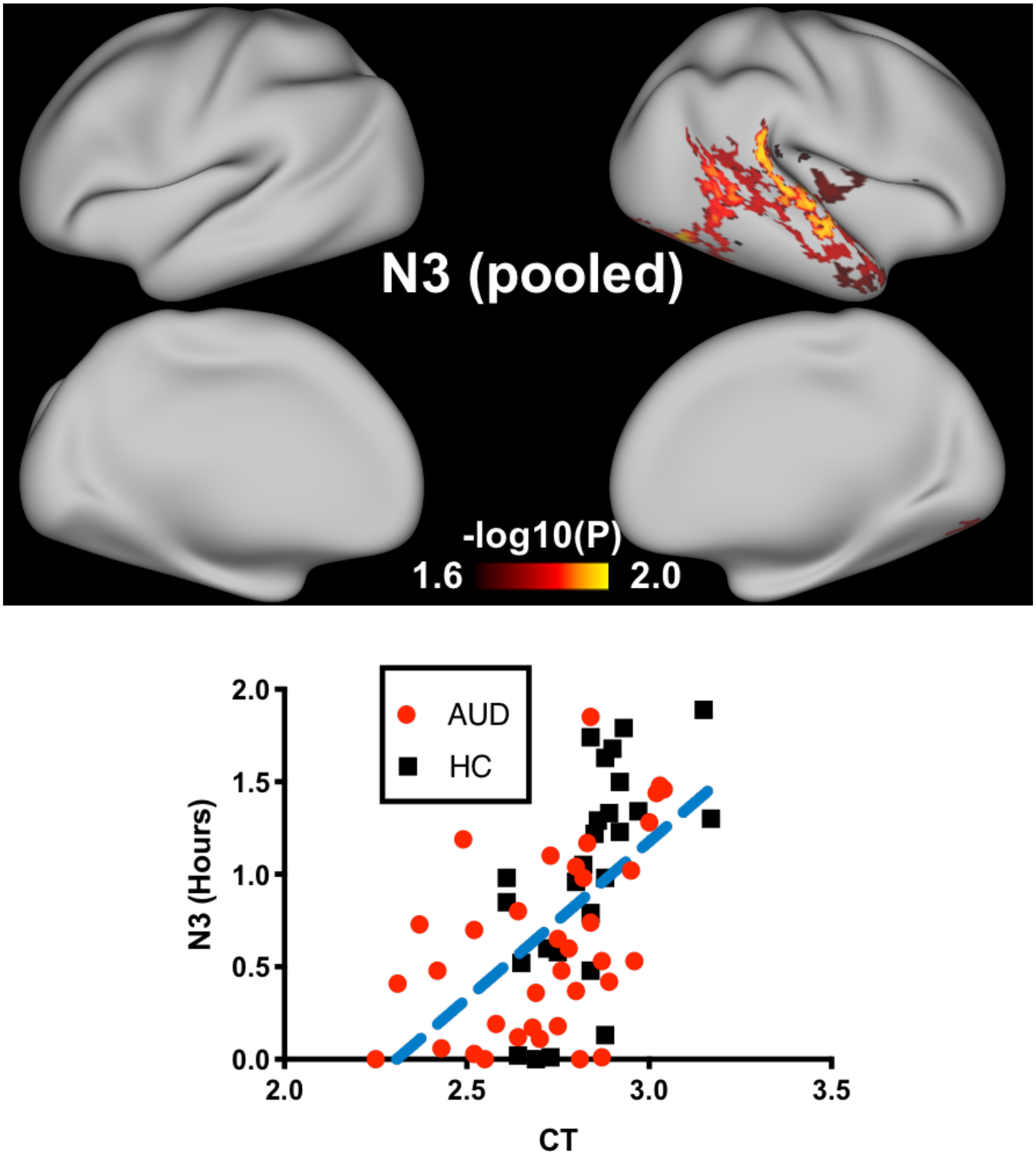

Figure S3 REM-GM association in AUD and HC pooled together

Left: REM sleep was associated with CT in the left motor areas, temporal, parietal and occipital cortex. The significance threshold was -log10(0.05/2) =1.602, Bonferroni-corrected for two hemispheres. **Right:** REM sleep was associated with GMD in cerebellum and left temporal pole (t-map). Color bar represents t values. The correlation between GMD (averaged across all the significant clusters) and REM duration was plotted to demonstrate the direction of the main effect.

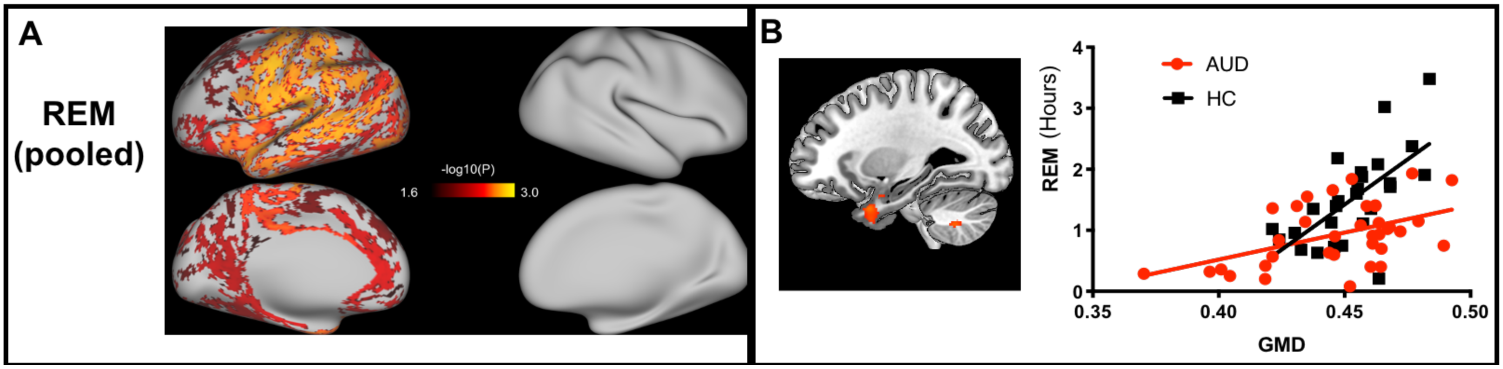

Figure S4 significant sleep-CT associations after controlling for age and gender

The significance threshold was -log10(0.05/2) =1.602, Bonferroni-corrected for two hemispheres.

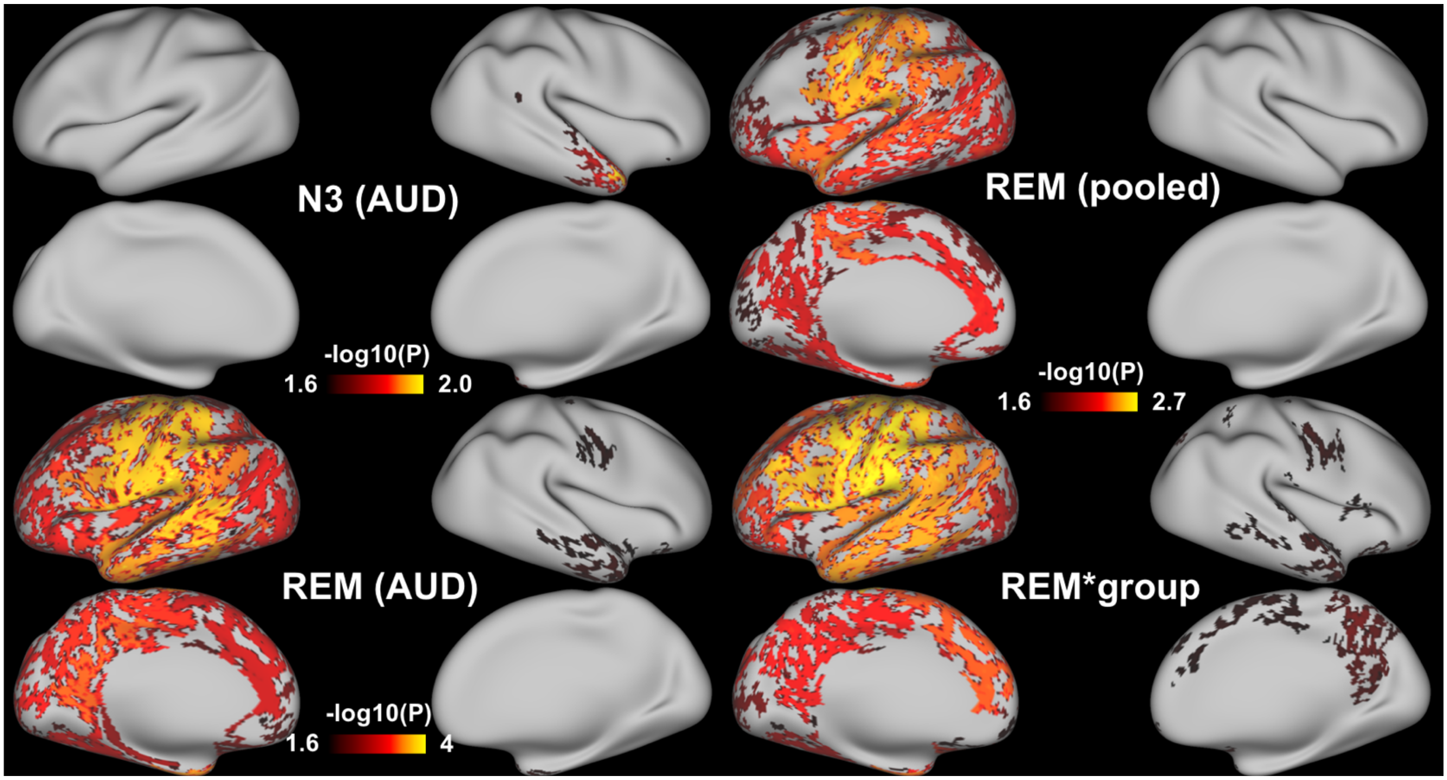

Results of ROI analyses for CT

Group comparisons for the structural brain measures (two-sample t-tests) revealed overall lower CT (46 out of 68 parcels) (FDR-corrected $p<.05$; **Table S1 and Figure S5a**).

For the combined group, TST was associated with overall CT for the regions that revealed group differences (all $r(62)>.262$, all FDR-adjusted $p<.05$) (**Table S2 and Figure S5b).** Longer N3 was strongly associated with greater CT in frontal, temporal and parietal areas and most prominent for the right hemisphere's rostral ACC, superior and inferior frontal gyrus, and superior and middle temporal gyrus (all $r(62)>.301$, all FDR adjusted $p<.05$) (**Table S2 and Figure S5c)**. Longer REM sleep was correlated with greater CT in a broad range of areas with the largest effect in left entorhinal and left rostral ACC (all $r(62)>.299$, all FDR adjusted $p<.05$) (**Table S2 and Figure S5d)**. In contrast to N3, REM had in general weaker association with CT especially for frontal areas and greater correlations with posterior regions including parietal and occipital gyri. No N2-correlated regions survived FDR correction**.** We also examined sleep-related CT variations separately in the two groups to control for correlations caused by group differences **(Table S2).** Interestingly, we found a positive correlation between REM and CT specially in the right lateral orbitofrontal, left supramarginal, temporal and motor areas (all $r(36)>.353$, all FDR adjusted $p<.05$) in AUD but not in HC **(Table S2 and Figure S5e)**. No other sleep-related CT variations survived FDR correction in either group.

Table S1 Group differences in cortical thickness (HC>AUD)

Parcels	T value	BH adjusted P
L-caudalmiddlefrontal	3.399	0
L-inferiorparietal	3.661	0
L-lateraloccipital	4.246	0
L-middletemporal	3.437	0
R-inferiorparietal	3.896	0
R-lateraloccipital	3.309	0
L-entorhinal	3.035	0.01
L-fusiform	2.798	0.01
L-inferiortemporal	3.068	0.01
L-rostralanteriorcingulate	2.774	0.01
L-superiorfrontal	3.193	0.01
L-supramarginal	3.104	0.01
R-caudalmiddlefrontal	3.093	0.01
R-fusiform	2.693	0.01
R-rostralmiddlefrontal	3.265	0.01
R-superiorfrontal	3.334	0.01
L-bankssts	2.596	0.02
L-lingual	2.316	0.02
L-paracentral	2.391	0.02
L-postcentral	2.419	0.02
L-precentral	2.511	0.02
L-precuneus	2.374	0.02
L-rostralmiddlefrontal	2.606	0.02
L-superiorparietal	2.333	0.02
L-superiortemporal	2.362	0.02
R-inferiortemporal	2.589	0.02
R-isthmuscingulate	2.407	0.02
R-middletemporal	2.661	0.02
R-posteriorcingulate	2.552	0.02
R-superiorparietal	2.493	0.02
R-superiortemporal	2.642	0.02
L-transversetemporal	2.312	0.03
R-parstriangularis	2.356	0.03
R-postcentral	2.238	0.03
R-precentral	2.525	0.03
R-rostralanteriorcingulate	2.36	0.03
R-supramarginal	2.228	0.03

L-frontalpole	2.222	0.04
L-insula	1.919	0.04
L-lateralorbitofrontal	1.954	0.04
L-parsopercularis	2	0.04
L-parsorbitalis	2.066	0.04
R-insula	2.41	0.04
R-cuneus	1.809	0.05
R-entorhinal	2.101	0.05
R-lateralorbitofrontal	2.345	0.05
R-parsopercularis	2.141	0.05
L-caudalanteriorcingulate	1.688	0.07
L-parstriangularis	1.679	0.07
L-posteriorcingulate	1.619	0.07
R-frontalpole	1.925	0.07
L-medialorbitofrontal	1.887	0.09
R-medialorbitofrontal	1.757	0.09
R-precuneus	1.629	0.09
L-parahippocampal	1.752	0.1
R-transversetemporal	1.739	0.1
R-bankssts	1.684	0.11
L-temporalpole	1.665	0.12
R-paracentral	1.645	0.12
L-cuneus	1.413	0.14
L-pericalcarine	1.375	0.16
R-temporalpole	1.349	0.16
R-parsorbitalis	1.362	0.17
L-isthmuscingulate	1.169	0.19
R-pericalcarine	1.099	0.24
R-caudalanteriorcingulate	0.654	0.45
R-parahippocampal	0.74	0.47
R-lingual	0.562	0.53

Table S2 Associations between sleep and cortical thickness in 68 parcels (FDR-corrected P<.05)

TST	r	N3	r	REM	
R_superiorfrontal	.429	R_rostralanteriorcingulate	.447	L_rostralanteriorcingulate	.359
R_inferiorparietal	.383	R_superiorfrontal	.438	L_entorhinal	.357
R_caudalmiddlefrontal	.380	R_parstriangularis	.413	L_bankssts	.353
L_superiorfrontal	.374	R_superiortemporal	.411	L_inferiortemporal	.353
R_rostralmiddlefrontal	.369	R_rostralmiddlefrontal	.390	L_inferiorparietal	.350
L_lateraloccipital	.366	R_caudalmiddlefrontal	.389	L_postcentral	.348
L_caudalmiddlefrontal	.359	R_middletemporal	.368	L_supramarginal	.348
L_middletemporal	.357	L_parsorbitalis	.333	L_lateraloccipital	.347
L_rostralanteriorcingulate	.351	L_superiorfrontal	.330	L_precentral	.338
L_entorhinal	.350	R_supramarginal	.314	L_middletemporal	.336
R_lateraloccipital	.344	R_lateralorbitofrontal	.312	R_lateraloccipital	.336
L_inferiorparietal	.341	R_fusiform	.311	L_insula	.328
L_supramarginal	.337	L_superiortemporal	.309	R_fusiform	.324
R_entorhinal	.330	L_frontalpole	.308	L_fusiform	.318
R_supramarginal	.330	R_inferiorparietal	.302	L_superiorparietal	.309
R_postcentral	.329	L_rostralmiddlefrontal	.301	R_entorhinal	.299
L_inferiortemporal	.322			R_lateralorbitofrontal	.299
R_fusiform	.321			**REM (AUD)**	
L_postcentral	.313			R_lateralorbitofrontal	.567
L_bankssts	.310			L_supramarginal	.558
R_inferiortemporal	.310			L_bankssts	.543
R_lateralorbitofrontal	.306			L_precentral	.530
R_parstriangularis	.303			L_middletemporal	.519
L_precentral	.301			L_postcentral	.518
L_fusiform	.294			L_inferiortemporal	.514
L_transversetemporal	.293			L_rostralanteriorcingulate	.479
R_superiorparietal	.292			L_superiortemporal	.478
R_superiortemporal	.292			L_entorhinal	.471
R_precentral	.290			L_transversetemporal	.463
R_insula	.289			L_paracentral	.454
L_insula	.288			L_inferiorparietal	.438
L_parsorbitalis	.287			R_superiortemporal	.433
L_paracentral	.284			L_superiorparietal	.430
L_superiorparietal	.281			L_precuneus	.428
R_parsopercularis	.281			L_insula	.424
R_middletemporal	.274			R_fusiform	.423
L_superiortemporal	.273			L_superiorfrontal	.421
R_isthmuscingulate	.268			L_fusiform	.418
L_precuneus	.262			L_parsopercularis	.409
L_rostralmiddlefrontal	.262			R_parsopercularis	.405
				R_precentral	.404
				L_lateralorbitofrontal	.400
				L_caudalmiddlefrontal	.396
				R_supramarginal	.396
				L_lateraloccipital	.392
				R_postcentral	.391
				L_parsorbitalis	.383
				R_inferiortemporal	.379

				L_rostralmiddlefrontal	.369
				R_superiorfrontal	.365
				R_middletemporal	.353

Figure S5 Sleep-CT associations (ROI analyses)

a) Group differences in CT (HC>AUD). Color bar represents effect size (Cohen's D) in 68 parcels from Desikan-Killiany cortical atlas. **b)** TST **c)** N3 and **d)** REM-related CT in AUD and HC pooled together and **e)** in AUD only**.** Color bars represent Pearson's r.

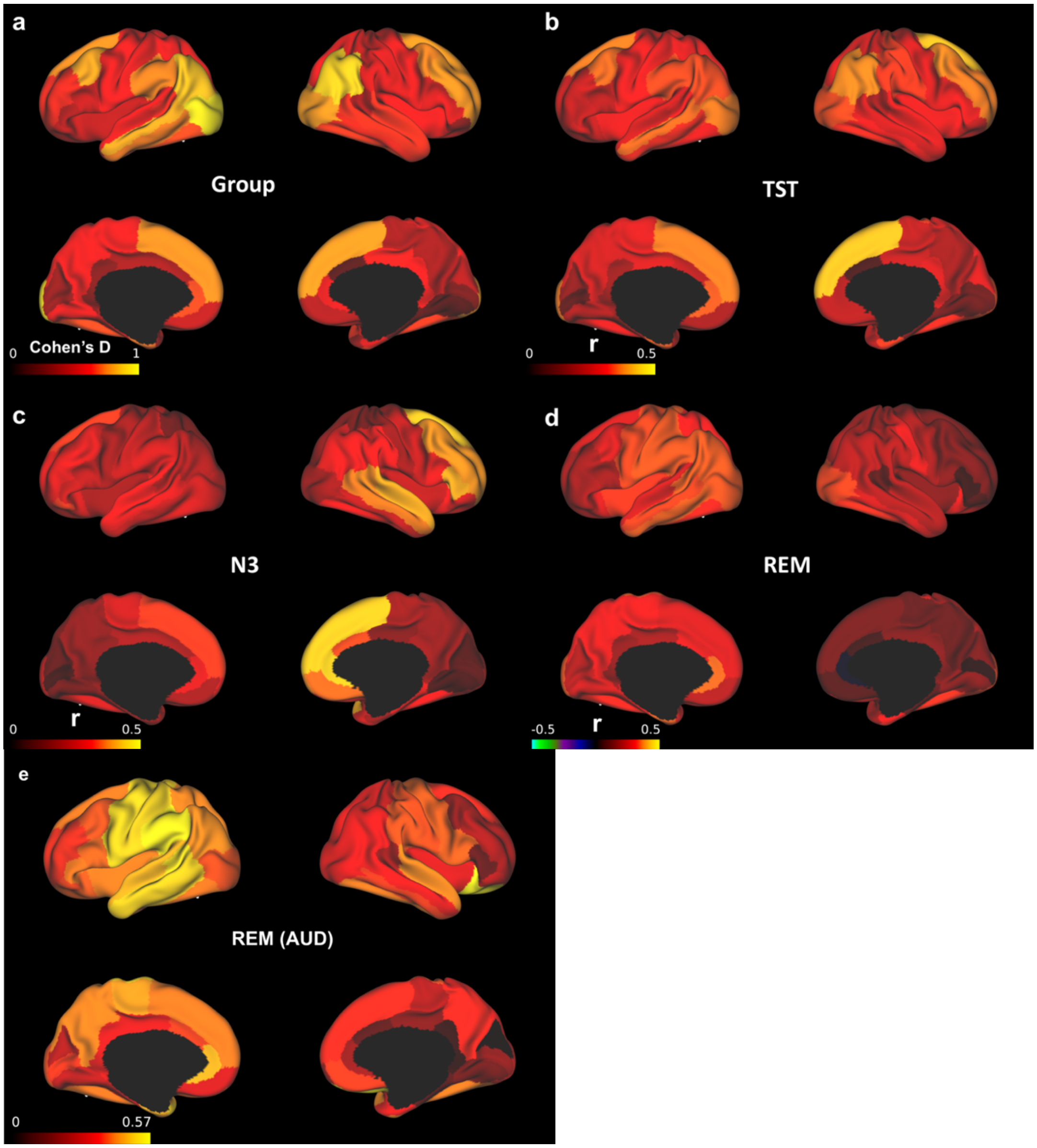

Correlation between two grey matter measures

CT was positively correlated with GMD in all regions except in left occipital cortex (all r>.265, p<.038) **(Figure S6)** though this correlation was driven by the AUD participants**.** When assessing each group separately, the positive correlations were only significant in AUD but not in HC (all r< .322, all p>.109) **(Figure S6)**.

Figure S6 Correlation between CT and GMD

The relationship between CT and GMD for different brain regions in AUD and HC pooled together **(left)**, AUD only **(middle)** and HC only **(right)**.

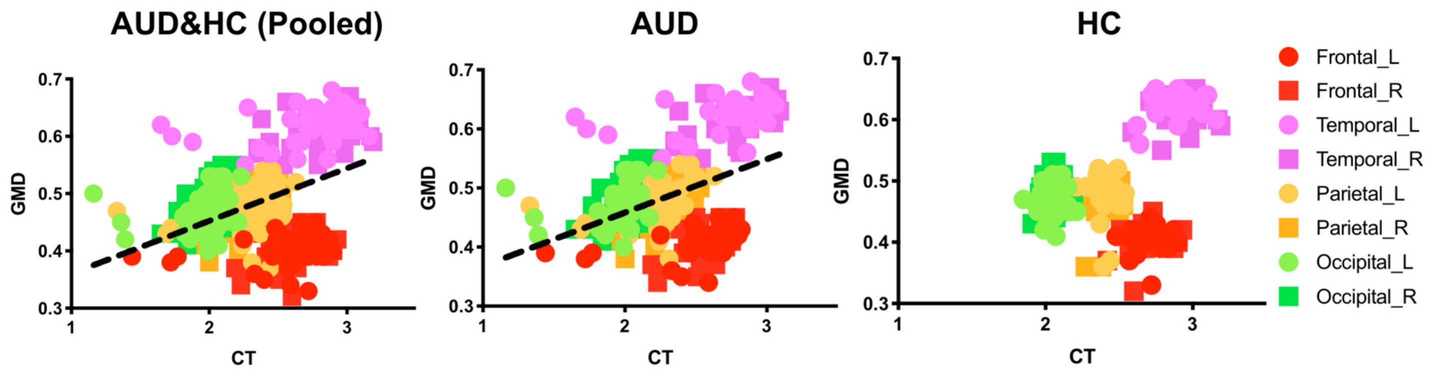

GM structure mediates the age*group interaction effect on REM sleep

Figure S7 Modelling

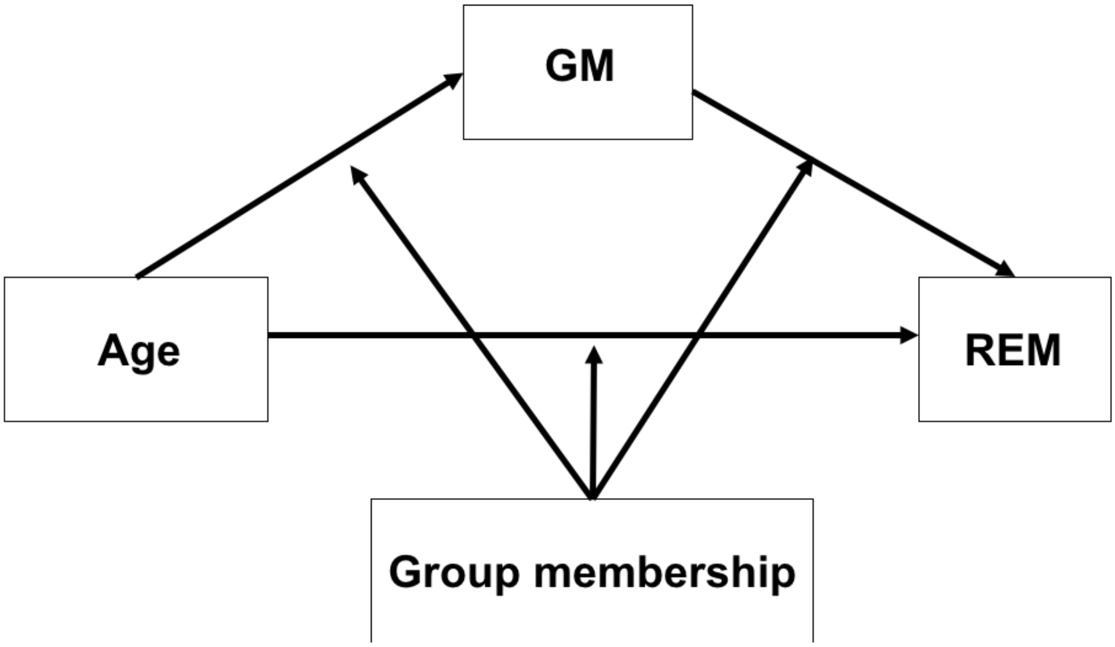

Methods

For these analyses we used averaged GMD and CT across the regions whose correlation with REM sleep differed between two groups.

Results

GMD

Aging was related to GMD reduction independent of group membership (t=-.37, p=.001). GMD, group and their interactions contributed to the duration of REM time (all |t|>2.35, all p<.05). After including the GMD as mediator and the group membership as moderator, the direct effect of age and age*group on REM sleep were not significant anymore (all |t|<.29, all p>.767). GMD mediated the age effect on REM in AUD (b=-.013, 95% CI [-.026, -.005]) but not HC (b=.019, 95% CI [-.002, .058]). The index of

moderated mediation (differences between conditional indirect effects) was statistically significant (Index= -.033, 95% CI [-.072, -.010]).

CT

There's a moderation effect of group on the correlation between age and CT (t=-2.29, p=.025). Age was negatively associated with CT in AUD (t=-5.15, p<.001) but not in HC (t=-1.33, p=.190). CT, group and their interactions contributed to the duration of REM time (all |t|>3.82, all p<.001). After including the CT as mediator and the group membership as moderator, the direct effect of age and age*group on REM sleep were not significant anymore (all |t|<.46, all p>.650). CT mediated the age effect on REM in AUD (b=-.015, 95% CI [-.030, -.004]) but not HC (b=.012, 95% CI [-.001, .035]). The index of moderated mediation (differences between conditional indirect effects) was statistically significant (Index= -.027, 95% CI [-.053, -.010]).

References

1. Arbanas G (2015): Diagnostic and statistical manual of mental disorders (DSM-5). *Alcoholism and Psychiatry Research; Zagreb* 51: 61–64.

2. Levendowski DJ, Ferini-Strambi L, Gamaldo C, Cetel M, Rosenberg R, Westbrook PR (2017): The Accuracy, Night-to-Night Variability, and Stability of Frontopolar Sleep Electroencephalography Biomarkers. *J Clin Sleep Med* 13: 791–803.

3. Berry RB, Brooks R, Gamaldo C, Harding SM, Lloyd RM, Quan SF, *et al.* (2017): AASM Scoring Manual Updates for 2017 (Version 2.4). *J Clin Sleep Med* 13: 665–666.

4. Finan PH, Richards JM, Gamaldo CE, Han D, Leoutsakos JM, Salas R, *et al.* (2016): Validation of a Wireless, Self-Application, Ambulatory Electroencephalographic Sleep Monitoring Device in Healthy Volunteers. *J Clin Sleep Med* 12: 1443–1451.

5. Lucey BP, Mcleland JS, Toedebusch CD, Boyd J, Morris JC, Landsness EC, *et al.* (2016): Comparison of a single-channel EEG sleep study to polysomnography. *J Sleep Res* 25: 625–635.
